# Supplementary material for: The influence of anthropogenic regulation and evaporite dissolution on earthquake-triggered ground failure
Source: Nat Commun. 2024 Mar 8;15:2114. doi: 10.1038/s41467-024-46335-3 (PMC10923926; doi:10.1038/s41467-024-46335-3)
Supplement: Supplementary file 1 — Supplementary Information [file 41467_2024_46335_MOESM1_ESM.pdf]

Supplementary Information for: The influence of anthropogenic regulation and evaporite dissolution on earthquake-triggered ground failure

Paula Bürgi[1] (pburgi@usgs.gov), Eric M. Thompson[1], Kate E. Allstadt[1], Kyle D. Murray[2], H. Benjamin Mason[1,3], Sean K. Ahdi[4,*], Devin Katzenstein[5,**]

[1] U.S. Geological Survey Geologic Hazards Science Center, Golden, CO, USA
[2] Department of Earth Sciences, University of Hawaii, Honolulu, HI, USA
[3] College of Engineering, Oregon State University, Corvallis, OR, USA
[4] AECOM, Los Angeles, CA, USA
[5] California Department of Transportation, Sacramento, CA, USA
[*] Previously at U.S. Geological Survey Geologic Hazards Science Center, Golden, CO, USA
[**] Previously at Searles Valley Minerals, Trona, CA, USA

Section S1: CPT-derived results

Cone penetration test (CPT) data are widely used in the geotechnical engineering community to map vertical soil profiles and assess soil properties with depth. CPT data are collected by mechanically pushing a rod into the subsurface and measuring the penetration resistance at the tip of the rod (which is usually shaped like a cone), i.e., cone resistance (q_c), and the friction of the materials passing by the cylindrical surface of the rod, i.e., sleeve friction (f_s). Discrete measurements of q_c and f_s are acquired at regular intervals; the sampling interval for all CPT profiles used in this study is 5 cm.

The first CPT-derived metric we compute is the soil behavior type index (I_c)[1]:

$$I_c = \sqrt{(3.47 - \log(Q_{t1}))^2 + (\log(F_r) + 1.22)^2} \qquad (1)$$

where Q_{t1} is the normalized cone resistance, and F_r is the normalized friction ratio where F_r is derived from the ratio of f_s and q_c. Both Q_{t1} and F_r are normalized by the total overburden stress at each depth interval. Robertson (2009, 2010) found that I_c correlates with the aggregate grain size within a given depth interval during which tip resistance and sleeve friction are measured[1,2]. Empirical studies[3] have found that I_c=2.6 is the approximate delineation between sand-dominated strata (I_c<2.6) and clay- and silt-dominated strata (I_c>2.6). It is also common to visualize the isoline where I_c=2.6 on a Q_{t1} versus F_r log-log graph, as shown in Figure 3c and Figures S1a & S1c.

The next metric we use is soil behavior type (SBT). In contrast to I_c, which is a continuous variable, SBT relies on discrete binning of $Q_{t1}$and F_r into “zones” that empirically correlate with distinct soil types and behaviors (description of zones provided in Table S1). SBT zones have been refined over the last few decades; here, we show two commonly used classifications[4,5]. SBT zones delineate boundaries between aggregate particle size as well as more specific

properties related to the expected behavior under cyclic stress caused by earthquakes. These include differences in density and plasticity[4] and expected volumetric changes[5] (i.e., contractive and dilative behavior). Contractive soils tend to contract (reduce in volume) under applied shear stress, leading to increased effective stress and a higher likelihood of liquefaction, while dilative soils tend to dilate (increase in volume) under applied shear stress, a soil behavior that usually protects against the triggering of liquefaction. Considering particle size and expected behavior together, the SBT zone where liquefaction is most likely to occur is sand-dominated and contractive, which correlates with Zone SC in Robertson (2016)[5].

Next, we compute the liquefaction potential index (LPI). LPI is a single, vertically integrated metric for a given CPT sounding (refer to Carlton et al., 2023 for a recent literature review on LPI[6]). This contrasts with the SBT and I_c metrics, which are calculated at each depth interval. Typically, there are tens to hundreds of intervals for an individual CPT sounding given the sampling rate of the CPT system. The derivation of LPI requires the calculation of an intermediary metric, the factor of safety (FS), which for most methods, requires f_s , q_c, water table depth, and the magnitude and peak ground acceleration of a given earthquake. Here, we present LPI results from two widely accepted formulations for FS[7,8]. LPI is then calculated using the following equation:

$$LPI = \int_{0\,m}^{20\,m} F\, w(z)\, dz \qquad (2)$$

where z is depth in meters, $w(z)$ is a depth weighting factor $w(z) = 10 - 0.5z$, and $F = 1 - FS$ if $FS \leq 1$, or $F = 0$ if $FS > 1$. LPI is considered only for a depth range between 0 and 20 meters because liquefaction is not expected to occur below 20 meters. LPI values can range from 0 (no expected liquefaction) to 100 (expected liquefaction). We calculate the LPI values for two FS formulations, presented in Table S2.

The final metric we use was developed by Geyin & Maurer (2020) and converts LPI to the probability of surface manifestation (F) reaching or exceeding a pre-defined manifestation severity (MSi)[9]:

$$F_{MSi}(LPI) = \Phi\left(\frac{ln(LPI) - ln(\theta)}{\beta}\right) \qquad (3)$$

where Φ is a Gaussian cumulative distribution function and θ and β are fragility function parameters derived in Geyin & Maurer (2020) for two liquefaction case-history inventories (Canterbury, New Zealand, and global) and for published FS formulations, including the two presented in Table S2 (i.e., Boulanger and Idriss, 2014[7]; Robertson and Wride, 1998[8]). In the model that uses the global inventory, the authors only derive fragility function parameters for the probability of any surface manifestation at all. In the model that uses the Canterbury, New Zealand, inventory, the authors parameterize for three scenarios, summarized here:

- Minor surface manifestation (less than 5% of ground surface is covered by ejecta; liquefaction features are generally isolated and smaller than a vehicle width);

- Moderate surface manifestation (between 5% and 40% of ground surface is covered by ejecta; liquefaction features are frequently adjoining and larger than a vehicle width); and
- Severe surface manifestation (more than 40% of ground surface is covered by ejecta; contiguous masses of liquefaction that are larger than a vehicle width).

In the study area with the hexagonal ejecta, 35% of the surface was covered with ejecta (at the upper end of “moderate” manifestation severity) and the size contiguous masses of liquefaction are much larger than a vehicle’s width; even the areas with relatively small ejecta features are typically on the order of 10–20 m wide. Although the parameters constrained for specific manifestation severities are derived from the Canterbury, New Zealand, inventory rather than the global inventory, it is worth noting that there is a <1% probability of severe surface manifestation. For the global inventory, which parameterizes for any manifestation at all, the probability remains low, on the order of 10–15%.

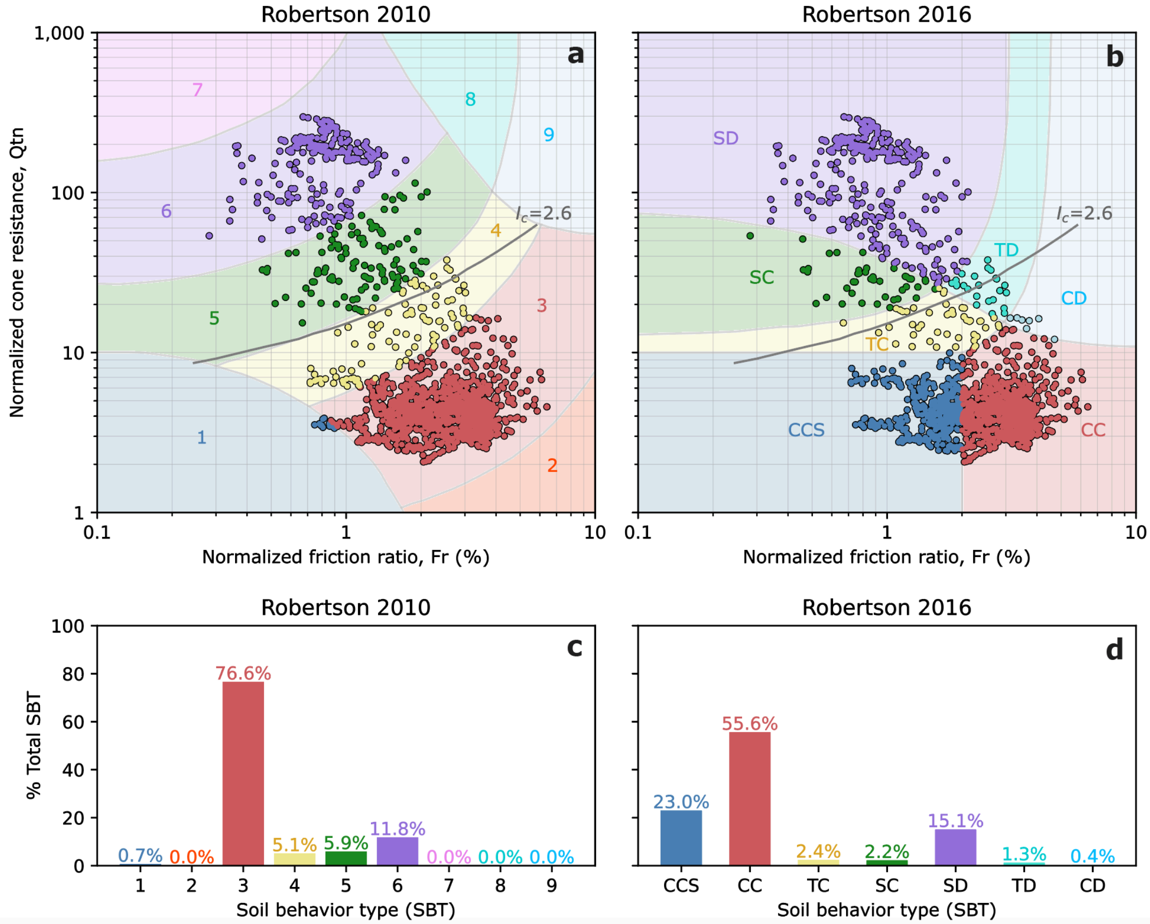

Figure S1: Additional CPT data and analysis. Data locations shown in Figure 1b, inset. CPT-derived analysis with I_c and soil behavior type delineations from: (a, c) Robertson (2010)[4], and (b, d) Robertson (2016)[5].

Table S1: SBT zones for Robertson 2010[4] versus Robertson 2016[5] shown in Figure S1.

Robertson (2010)[4]		Robertson (2016)[5]	
Zone	***SBT***	***Zone***	***SBT***
1	Sensitive fine-grained	CCS	Clay-like – Contractive - Sensitive
2	Clay - organic soil	CC	Clay-like – Contractive
3	Clays: clay to silty clay		
4	Silt mixtures: clayey silt & silty clay	TC	Transitional – Contractive
5	Sand mixtures: silty sand to sandy silt	SC	Sand-like – Contractive
6	Sands: clean sands to silty sands	SD	Sand-like – Dilative
7	Dense sands to gravelly sand		
8	Stiff sand to clayey sand	TD	Transitional – Dilative
9	Stiff fine-grained	CD	Clay-like – Dilative

Table S2: Mean LPI and mean probability of liquefaction surface manifestation derived from the seven CPT soundings presented in the "geotechnical analysis" section of the main text, and locations shown in Figure 1b.

<table>
<tr><td rowspan="2"></td><td></td><td colspan="3">Probability of manifestation
(Geyin & Maurer, 2020)[9]</td></tr>
<tr><td>Mean LPI</td><td colspan="2">Canterbury, NZ model</td><td>Global model
(any manifestation)</td></tr>
<tr><td rowspan="3">FS model (Robertson & Wride, 1998)[8]</td><td rowspan="3">1.14</td><td>Minor</td><td>31.4%</td><td rowspan="3">11.3%</td></tr>
<tr><td>Moderate</td><td>10.3%</td></tr>
<tr><td>Severe</td><td>0.2%</td></tr>
<tr><td rowspan="3">FS model (Boulanger & Idriss, 2014)[7]</td><td rowspan="3">1.86</td><td>Minor</td><td>23.1%</td><td rowspan="3">14.6%</td></tr>
<tr><td>Moderate</td><td>4.4%</td></tr>
<tr><td>Severe</td><td>0.1%</td></tr>
</table>

Section S2: Field-observed subsidence of a Searles Lake solution mining injection well

In March 2023, authors of this study visited the Searles Lake study area including the locations of several injection and production wells that were drilled after the Ridgecrest earthquake. When an injection well is drilled, an L-shaped bracket is sometimes welded onto the side of the well shaft such that the bottom of the bracket is approximately at ground level. Thus, the bracket can be used as an approximate measure of ground subsidence over time by measuring the vertical distance from the ground surface to the bracket bottom. For the well featured in Figure S2c-d, we measured 9 cm of subsidence between January 2021 (when well drilling began) to March 2023 (date of fieldwork). Our field measurement of 9 cm of subsidence is very approximate; the ground was uneven, and a single ground "surface" was not obvious at the time of our fieldwork and (presumably) at the time of the bracket emplacement. However, our field observations confirm that subsidence occurs at injection well sites.

The Interferometric Synthetic Aperture Radar (InSAR) time series for the well pictured in Figure S2c-d is shown in Figure S2e. The area where most of the post-earthquake wells are located has InSAR displacement contributions that are not related to the drilling, resulting in spatial heterogeneity in the InSAR data and making it difficult to compare the signal at this injection well with pixels in farther away, unmined areas. Thus, we compare the time series of the injection well pixel (colored red) with a ring of pixels within ~50 m of the injection well pixel (i.e., pixels between the injection well pixel and adjacent wells, colored beige). The difference between the red and beige lines after the well drill date (January 2021) represents the well subsidence over time. The cumulative difference is approximately 1–2 cm during the observation period. This is a much smaller measurement of subsidence than the 9 cm observed in the field. However, it is important to point out several caveats that make it difficult to directly compare the InSAR observations with the field measurements. First, we do not know how broad or localized the injection well-related subsidence is (i.e., the extent to which the subsidence signal "bleeds over" into adjacent pixels). Thus, the divergence of the pixel containing the injection well (Figure S2b, red) and the surrounding pixels (Figure S2b, beige) can only represent a minimum threshold of subsidence. Also, the InSAR time series does not extend to the date of the field visit, and we do not know how much more subsidence accrued between the end of the InSAR time series and the date of the field visit. Considering these factors, we only use this information to confirm that some degree of subsidence is observed at injection wells in two independent measurement techniques.

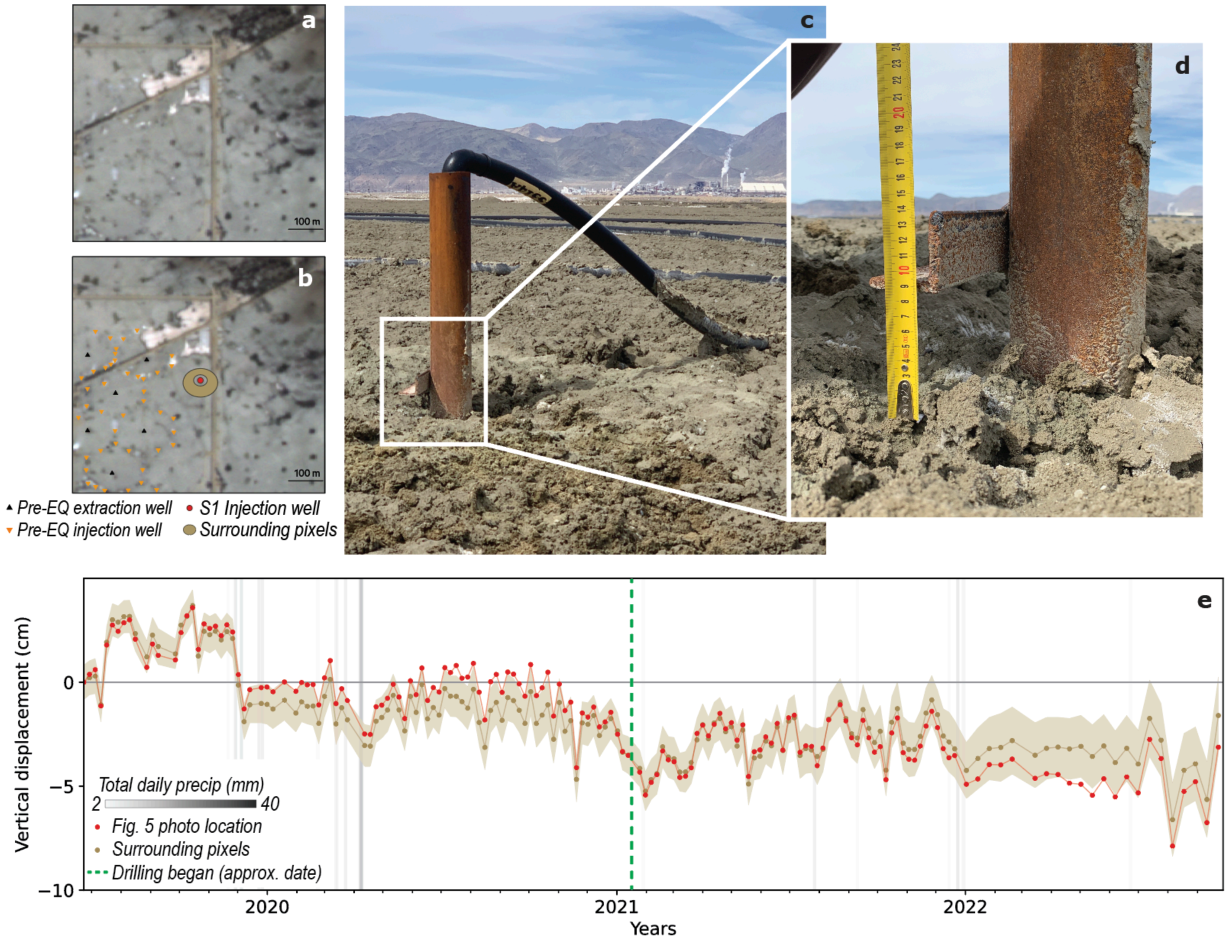

Figure S2: Field-observed versus InSAR-observed subsidence of an injection well drilled post-earthquake. (a) Unannotated and (b) annotated optical imagery acquired 09/27/2022 (© 2022 Planet Labs PBC) showing the location of the photographs and time series pixels in (c)-(e). (c) and (d) Photographs of a solution mining fluid injection well in Searles Lake (photo credit: P. Bürgi). The well was drilled in January 2021, and these photographs were taken in March 2023. The L-bracket attached to the side of the injection well was at ground level when the well was drilled in January 2021. Since being drilled, the well has subsided approximately 9 cm. (e) Post-earthquake InSAR time series of the pixel containing the injection well shown in (c) and (d) (red) and the pixels within ~50 m of the pixel containing the injection well (beige) where the points represent the mean, and the shaded area represents the standard deviation pixels in the beige area. The green dashed line indicates the approximate date that drilling began for this injection well.

Any use of trade, firm, or product names is for descriptive purposes only and does not imply endorsement by the U.S. Government.

References

1. Robertson, P. K. Interpretation of Cone Penetration Tests — A Unified Approach. *Can. Geotech. J.* **46**, 1337–1355 (2009).
2. Robertson, P. K. Evaluation of Flow Liquefaction and Liquefied Strength Using the Cone Penetration Test. *J. Geotech. Geoenvironmental Eng.* **136**, 842–853 (2010).
3. Idriss, I. M. & Boulanger, R. W. *Soil Liquefaction During Earthquakes*. (Earthquake Engineering Research Institute, 2008).
4. Robertson, P. K. Soil Behaviour Type from the CPT: An Update. in *2nd International Symposium on Cone Penetration Testing* vol. 2 (2010).
5. Robertson, P. K. Cone Penetration Test (CPT)-based Soil Behaviour Type (SBT) Classification System — An Update. *Can. Geotech. J.* **53**, 1910–1927 (2016).
6. Carlton, B., Geyin, M. & Engin, H. K. Evaluation of Case-based Reasoning to Estimate Liquefaction Manifestation. *Earthq. Spectra* (2023) doi:10.1177/87552930231203573.
7. Boulanger, R. W. & Idriss, I. M. CPT and SPT Based Liquefaction Triggering Procedures. *Cent. Geotech. Model.* (2014).
8. Robertson, P. K. & Wride, C. (Fear). Evaluating Cyclic Liquefaction Potential Using the Cone Penetration Test. *Can. Geotech. J.* **35**, 442–459 (1998).
9. Geyin, M. & Maurer, B. W. Fragility Functions for Liquefaction-Induced Ground Failure. *J. Geotech. Geoenvironmental Eng.* **146**, (2020).
